# Factors Associated with Missed Vaccination during Mass Immunization Campaigns

**DOI:** 10.3329/jhpn.v27i3.3378

**Published:** 2009-06

**Authors:** William M. Weiss, Peter J. Winch, Gilbert Burnham

**Affiliations:** ^1^ Health Systems Program, Department of International Health, Johns Hopkins Bloomberg School of Public Health, 615 North Wolfe Street, Room E8132, Baltimore, MD 21205, USA; ^2^ Social and Behavioral Interventions, Department of International Health, Johns Hopkins Bloomberg School of Public Health, 615 North Wolfe Street, Room E8132, Baltimore, MD 21205, USA

**Keywords:** Child health, Evaluation studies, Immunization, Polio, Polio vaccine, Review literature, Vaccine

## Abstract

Achieving a high percentage of vaccination coverage with polio vaccine, while necessary, is not sufficient to eliminate or eradicate polio. The existence of pockets of under-vaccinated children has allowed outbreaks of polio in countries that have achieved high levels of vaccination coverage and in countries with no cases for many years. In a literature review, 35 articles were identified that described factors associated with missed vaccination in mass immunization campaigns. An annotated bibliography was developed for each article; these were then coded using the AnSWR program, and codes were organized into three larger thematic categories. These thematic areas were: (a) organization and implementation of mass campaigns; (b) population characteristics; and (c) knowledge and practices of caretakers. If these factors were geographically clustered, it was suspected that these clusters might have higher likelihood of becoming pockets of unvaccinated children. Immunization programme managers can target resources to identify if such clusters exist. If so, they can then ensure supervision of vaccination efforts in those sites and take further action, if indicated, to prevent or mitigate pockets of unvaccinated children.

## INTRODUCTION

At the end of 2007, four countries—Afghanistan, India, Nigeria, and Pakistan—were endemic for polio (1). There were fewer (1,303) polio cases in 2007 than in 2006 or 2005 (1,979 and 1,997 respectively); fewer (n=12) countries had polio transmission than since 2002 when nine countries had polio transmission (2). The four polio-endemic countries accounted for 92% of cases in 2007, with India and Nigeria reporting, respectively, 66% and 22% of all cases in the world (1). Of the two types (WPV1 and WPV3) of wild polio remaining in circulation, the number of WPV1 cases decreased in 2007 by 81% compared to 2006 (2). The decrease in WPV1 was attributed to the priority given to eradication of WPV1—the most virulent type of wild polio (3). Priority attention was given to WPV1 through widespread use during mass immunization campaigns of monovalent oral polio vaccine (mOPV1) for WPV1. This vaccine (mOPV1) was used in place of the most commonly-used vaccine—trivalent OPV (tOPV) because it is more effective at interrupting the transmission of WPV1 than tOPV. It does not, however, provide protection against WPV3 (3). The extensive use of mOPV1 during mass immunization campaigns in 2007 may have allowed for the approximate tripling of the number of WPV3 cases compared to 2006, especially in India with 77% of WPV3 cases in the world in 2007 (2-4).

The current strategy for interrupting the transmission of poliovirus includes the folowing five elements: (a) intensified mass immunization campaigns in endemic areas; (b) response to outbreak in countries that are polio-free or with importations, as needed; (c) mass immunization campaigns in countries with importations and countries at the highest risk of importation; (d) improvement of routine immunization coverage against polio; and (e) high-quality surveillance and laboratories (3,5). The first three of the five elements rely on mass immunization campaigns, with much of the effort to be focused on specific geographic areas. In 2008, the priority for mass immunization campaigns continues to eradicate WPV1 using mOPV1, with some use of mOPV3 and tOPV to suppress WPV3 until WVP1 is eradicated (3). Maintaining or improving the quality of mass immunization campaigns is critical to the success of these efforts.

Mass immunization campaigns—most often launched as a strategy to eradicate polio and increasingly being used for other diseases—frequently achieve very high levels of vaccination coverage; coverage rates of 80-90% of the target population are common (6-13). Achieving a high percentage of vaccination coverage, while common and necessary, may not be sufficient to eliminate or eradicate polio. The literature provides a number of examples where outbreaks of the disease occurred in under-immunized populations living within otherwise highly-immunized populations (14-20).

Because outbreaks of the disease have occurred in populations with high immunization coverage, information about the coverage levels in the general population is not sufficient to determine whether a population is at risk for importation or transmission of polio into new areas. This suggests that it is vital to know whether pockets of under-vaccinated children exist in an otherwise highly-immunized population. We define the term ‘pocket’ as a group of unvaccinated persons who are located closer together geographically than would be the case if these persons were distributed randomly in a population of interest. While it is desirable to know whether pockets of unvaccinated persons exist, it is not clear how best to locate these pockets in advance.

The purpose of this paper was to identify, through literature review, factors associated with who is missed in a mass campaign. This is an important first step in the process of identifying potential pockets of unvaccinated persons. Then, if one or more of these associated factors are known to be clustered in a geographically-focused site within a larger programme area, we may consider this site as having a higher likelihood of being or becoming a pocket of unvaccinated persons. Understanding such factors and then how they are distributed can help us predict if and where potential pockets of unvaccinated persons might exist in a population. If potential pockets of unvaccinated persons are suspected, we can take additional steps before, during, and after a mass vaccination campaign to verify, prevent and/or address this potential problem.

## MATERIALS AND METHODS

A literature search was made using MEDLINE and ANTHROSOURCE to identify relevant studies and articles. The literature search used combinations of the following Medical Subject Headings (MeSH) from the National Library of Medicine: program evaluation; evaluation-studies; immunization; and polio. These search headings were combined with the following keywords for additional searches: mass; campaign; and vaccination. Abstracts of 34 articles found through this search were reviewed for relevance. The majority of the articles selected were evaluations of mass campaigns for polio. Several articles that discussed mass campaigns for measles and other vaccines were also deemed relevant to this paper and were included. The most common method used in these articles to evaluate campaigns was a household sample survey followed by surveillance data. A final set of articles, identified through this process, was obtained from libraries at the Johns Hopkins University or through online services. In addition to these sources, the author received an unpublished report of mass campaign evaluations from field staff of polio-eradicationprojects of the CORE Group's Polio Eradication Partners Project funded by the US Agency for International Development (http://www.coregroup.org/initiatives/polio.cfm). The report documented the vaccination coverage in programme areas following mass campaigns and investigated reasons why some children remained unvaccinated.

An annotated bibliography was developed listing the citation of each article. Under each citation, we listed the key factors cited in the article associated with whether or not a person in the target population received vaccine during the mass campaign being studied. We identified 19 key factors and developed a code for each. The annotated bibliography was then coded using the AnSWR program developed by the US Centers for Disease Control and Prevention for analysis of qualitative or word-based data (21). Codes were then organized into thematic categories. We then developed sublists of citations by category and code.

## RESULTS

In reviewing and coding the 35 articles and the report identified through searches, three thematic areas emerged from the literature review of the factors associated with vaccination status following a mass vaccination campaign. The themes are mentioned individually. However, these themes (and the factors identified within each) do not necessarily operate independently and do overlap. The three themes are the following: (a) factors relating to organization and implementation of mass campaigns; (b) factors relating to population characteristics of unvaccinated children; and, (c) factors relating to knowledge and practices of caretakers. The results of the literature review are presented below by each of these topical or thematic areas.

### Factors relating to organization and implementation of mass campaigns

Experiences with elimination of polio in the Western Pacific regions suggest that there will be more unvaccinated children where the planning phase at the district level and below was poor (22,23). For example, Bilous *et al*. found that countries in the Western Pacific region that had the greatest success in mass campaigns were those where logistics spreadsheets were developed down to the district level, and hand-made maps were drawn at the health centre level (22).

The unwillingness of health workers or authorities to vaccinate due to concerns about safety of vaccine was associated with many unvaccinated persons during mass campaigns. For example, health authorities in several states of northern Nigeria suspended mass campaigns for polio in 2003 and 2004 citing concerns with the safety of the vaccine (24,25). This resulted in pockets of large populations with unvaccinated children allowing continued transmission and resulting in importation of wild polio into 10 polio-free countries in West, Central, South and North Africa. A study of health workers who participated in a mass polio-vaccination campaign in 2003 in Gombe, Nigeria, found that some health workers believed that the polio vaccine caused sterility, contained the HIV virus, and/or was harmful if given to a child repeatedly (26).

The unwillingness or inability of health workers to vaccinate due to security concerns is another reason that people remain unvaccinated. In another example in Angola, because of security concerns due to the civil war, 51 municipalities in 1999, 24 in 2000, and 10 in 2001 were not provided vaccination services during at least one mass polio campaign (27). In 2004, the performance of mass campaigns has worsened in southern and southeastern Afghanistan because of continued poor access to insecure areas along the border with Pakistan (28).

Experience with elimination of polio in the Americas suggest that there will be more unvaccinated persons following mass campaigns in areas where fixed-post vaccination was carried out compared to areas that were covered by house-to-house vaccination. According to de Quadros, “countries such as Mexico, Brazil, Columbia, and Peru that employed national vaccination days without the house-to-house component continued to have outbreaks of polio … it became apparent that despite the two strategies (routine delivery and ‘fixed post’ national vaccination days), efforts were not reaching all pockets of children, and wild poliovirus transmission remained unchecked” (29). Note that problems can still be found in areas where house-to-house vaccinations were carried out. In Pakistan, for example, unvaccinated children were more likely to have caretakers who reported that the mobile team did not come to the home as planned (6). In India, unvaccinated children were more likely to live in households served by house-to-house vaccination teams who were under-supervised and failed to carry out key tasks, or where a vaccination team came to the house when a parent and/or the child was not at home (30).

Several studies highlight the influence of media and information on the results of mass campaigns. Following a mass campaign in Pakistan, unvaccinated children were more likely to live in homes without a TV or a radio, or have a caretaker who reported not being informed at least one day in advance of the campaign (6). In Egypt, children missed by mass campaigns were more likely to have a caretaker who did not watch TV, who reported having a limited number of information sources, or who reported not being informed at least one day in advance of the campaign (31). Lin *et al*. found that, following a mass campaign in El Salvador, unvaccinated children were more likely to have a caretaker who did not listen to the radio on the day before the survey, did not read newspapers, or reported being informed about the campaign too late (32).

A personal invitation to participate in a mass campaign also appears to affect the results (22). In Mexico, unvaccinated children were more likely to have caretakers who were not personally invited to participate in the mass campaign; either health workers or volunteers did not visit mothers in their homes or solicit participation of mothers as they visited or passed by immunization health posts (33). In El Salvador, children vaccinated during a mass campaign were more likely to have caretakers informed by a ‘local disseminator’ (32). Gomber *et al*., following a study of mass campaign awareness in India, determined that the use of personal invitations to caretakers could overcome a lack of awareness about the campaign (7). These findings are supported by a study in India, which found that children who were enumerated prior to the mass campaign were more likely to be vaccinated during the campaign; enumeration can serve as a personal invitation (8).

Experience in Cuba suggests that remarkable success of the country in eliminating polio via mass campaigns was due, in part, to the involvement of grassroots groups in social mobilization during a crisis in the health system (1962-1967) when 50% of physicians left the country (34). By implication, we might expect that unvaccinated children are more likely to live in areas without the involvement of grassroots group in social mobilization for mass campaigns.

Other things governments can do to prevent missing persons during mass campaigns are to provide certain kinds of incentives to participate. Results of focus-group discussions with caretakers following mass campaigns in Uganda suggest that providing a campaign-specific vaccination card to caretakers documenting receipt of vaccine would be a motivating incentive to participate and perhaps the single most important solution for increasing coverage during campaigns (35,36).

### Factors relating to population characteristics of unvaccinated children

Migration due to economic crises or conflict is a risk factor for persons being missed by a mass campaign, especially recent arrivals to urban areas or refugee camps. Evaluations of mass campaigns in South Africa have found that unvaccinated urban children were more likely to have been recent in-migrants (37,38). An evaluation of a mass vaccination campaign in a Macedonian refugee camp for Kosovar Albanians found that unvaccinated children were more likely to have been recent arrivals to the camp (39). Results of another study of mass vaccination campaigns among several Macedonian refugee camps showed that the coverage was the lowest in those camps experiencing the highest rate of population turnover (40). Experience of elimination of polio from the Americas also suggests that unvaccinated children are more likely to live in areas with heavy migration (29). A study in Columbia found that children living in areas with a high proportion of displaced families were more likely to be unvaccinated following a national immunization day (41).

The picture of whether urban people are at a higher risk for being missed by mass campaigns is not entirely clear. Several studies have found that mass immunization campaigns tend to miss persons living in rural or ‘remote’ urban areas (42,43). In contrast, Lewis *et al.* found that a mass campaign in Ukraine was more likely to miss in urban people than rural people (44). What appears to be clearer is that slum children are at a higher risk of being missed by mass campaigns than children living in other urban or rural areas (11,45,46). Evaluations of mass campaigns and reviews of polio-eradication progress also cited living standards as a risk factor for being missed by mass campaigns. Experience with elimination of polio in the Americas suggest that those living in poverty had a higher risk of being missed (29,33) as do those living in homes without brick, tile, or cement floors (32).

In some countries, minority ethnic or religious affiliation was associated with vaccination status during mass campaigns. For example, results of an evaluation of a mass campaign in India showed that unvaccinated children were more likely to be Muslims than Hindus, although Muslims represent a smaller proportion of the population than Hindus (9). Minorities may be suspicious about intentions of health providers and refuse services, or health providers may neglect in providing services to minorities, or both. Another study in India found that, while mass campaigns reduced inequities in coverage due to gender, caste, or wealth status, inequities in coverage associated with religion remained (47).

Where vaccination is provided from a fixed-post during mass campaigns, distance can affect those who receive vaccine and who do not. Results of evaluations of mass campaigns showed that children living more distant from posts were less likely to be vaccinated in Ghana (48), Egypt (31), and Pakistan (6).

The World Health Organization (WHO) recommends that mass campaigns for the elimination of polio provide OPV to all children aged less than five years. Results of numerous evaluations of mass campaigns have also shown that the campaigns are less likely to vaccinate either the youngest or the oldest age-groups within the under-five population. In Pakistan, Egypt, and India, unvaccinated children were more likely to be aged less than one year (6,9,24,31,45). In Egypt and India, children aged over three years were also more likely not to have received vaccination during a mass campaign (11,31).

### Factors relating to knowledge and practices of caretakers

The knowledge of caretakers appears to have great influence on who is and who is not vaccinated during mass campaigns. Being unaware of the campaign was the most frequently-cited reason given by caretakers why their children were not vaccinated across a range of studies in Egypt (31), El Salvador (32), Ghana (48), India (7-9,24,45,49), Mexico (33), and Pakistan (6).

Caretakers who had incorrect knowledge about vaccines or mass campaigns were less likely to have their children vaccinated during a mass campaign. Caretakers may be unaware of the benefits of polio vaccine. For example, a study of mass polio-immunization campaigns in December 2001 and January 2002 in Chad found that the proportion of unvaccinated children was higher among caretakers who did know the benefits of polio vaccine (43). On the other hand, caretakers may have mistaken knowledge about the timing, number of doses needed for immunity, or side-effects. In an evaluation of a campaign in Mexico, unvaccinated children were more likely to have caretakers who decided not to vaccinate because the child had ‘already been vaccinated’ or because they thought that an immunization card was a prerequisite to have their children vaccinated or because the child was ill (33). Reichler *et al.* found that children who were sick during a campaign in Pakistan were less likely to be vaccinated (6). Mukherjee and Ghose, and Jajoo *et al.* found that unvaccinated children in a mass campaign in India were more likely to have caretakers who stated concerns about sideeffects of OPV (30,45). In another Indian study, caretakers of unvaccinated children stated that there was no need for more doses, but that three doses prior to the campaign were sufficient (49). Studies in Uganda found that caretakers of unvaccinated children thought that a previous national immunization day had caused an epidemic of malaria that killed a large number of children (36). In Ukraine, Lewis *et al.* found that the unvaccinated were more likely to think that the vaccine was ineffective or unsafe or that a booster dose was not needed (44). In Cameroon, a rumor that tetanus toxoid vaccination campaign was being used by public-health workers to sterilize women led some women to run away from vaccination teams and eventually led to cancellation of the campaign (50).

The caretaker being busy was another reason that caretakers report why their children were not vaccinated during a mass campaign. This reason was cited in evaluation studies in Ghana, India, and Pakistan (6,8,30,48).

Having a caretaker who cannot read also appeared to be a risk factor for failure in being vaccinated during a mass campaign. Three studies in India found that children with illiterate mothers and/or fathers were more likely to be missed during a mass campaign (9,12,45).

## DISCUSSION

Mass campaigns are considered an essential element in the effort to interrupt the transmission of vaccine-preventable diseases and should, therefore, be assessed for quality-assurance purposes. In special studies that assessed the vaccination coverage of a mass campaign using population-based coverage surveys, high levels of coverage were common. Achieving high overall coverage appears insufficient to eliminate or eradicate some diseases. The existence of pockets of under-vaccinated children within areas with high coverage has allowed outbreaks of measles. For this reason, assessments of vaccination coverage for the general population may not be sufficient; information about existence of pockets of under-vaccinated persons is also important.

In this paper, we have identified the following factors associated with who is missed by a mass campaign, even those campaigns with high population levels of coverage: (a) factors relating to organization and implementation of mass campaigns; (b) factors relating to population characteristics of unvaccinated children; and (c) factors relating to knowledge, attitudes, and practices of caretakers. A conceptual framework describing how these factors may lead to a pocket of unvaccinated persons and its consequences is provided in the figure. The conceptual framework in the figure is an adaptation of the PRECEDE Model for health-promotion planning based on the factors discussed above (51). In this figure, we use the example of polio because most literature available studied this disease; we believe this model can be used for other diseases addressed by mass campaigns.

The PRECEDE Model is designed to be reviewed from right to left. The model begins with an Epidemiological Diagnosis that describes the health problem. The next phase is the Behavioural and Environmental Diagnosis that describes (a) behavioural and (b) non-behavioural factors directly leading to the health problem being analyzed. The term Environmental Factors in this model is a broad term to include the total context in which the specified behaviour occurs and that influences the health problem being analyzed. Environmental factors may also influence the health behaviour directly. The next phase of the model (Educational and Organizational Diagnosis) describes the underlying factors that influence the factors identified in the Behavioural and Environmental Diagnosis. Any of these factors may also influence each other and may influence both behavioural and environmental factors.

**Fig.**
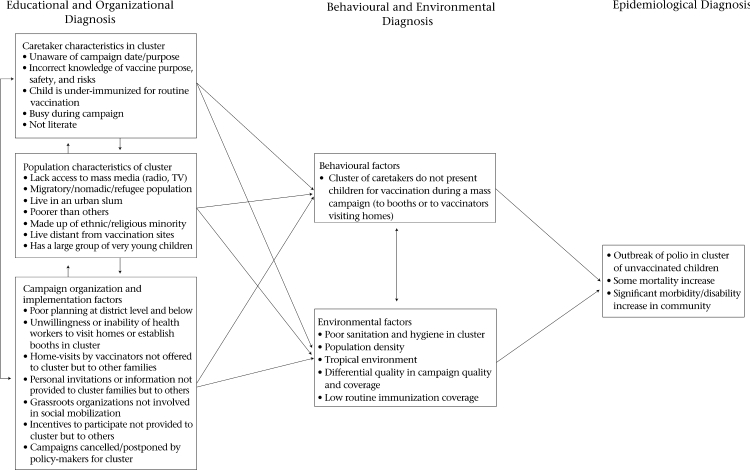
Conceptual framework—origins of a pocket of unvaccinated persons following a mass immunization campaign (example of polio) based on potential causal factors identified through a literature review.

In this adapted model, an outbreak of polio occurs, along with its consequences, when the virus enters a cluster of children who remain susceptible in an otherwise highly-immunized population. This is described in the model under the Epidemiological Diagnosis phase. One advantage of the PRECEDE model is that it allows us to hypothesize about multiple types of factors that lead to a cluster of children being unvaccinated. And although there is a behavioural component in the model—children in a cluster not receiving vaccine—, the model avoids improperly assuming that the caretakers or families of children are to blame for this. The model forces us to look at the larger environmental and structural context, including the health system, in which this problem occurs. This is described in the Behavioural and Environmental Diagnosis.

The environmental diagnosis here describes factors that may lead to an outbreak of polio directly, or factors that may indirectly lead to an outbreak by affecting the health behaviour specified. In addition to the physical environment, such as poor sanitation and hygiene in a cluster and a tropical environment, lowering efficacy of vaccines for enteric diseases, environmental factors may also include the quality of campaigns and the levels of routine immunization coverage.

Another advantage of the model is that it asks the user to develop hypotheses about the underlying reasons for the behavioural and environmental factors listed in the model. This is the Educational and Organizational Diagnosis phase of the model. There are many factors that influence behaviour or the physical and social environment. These factors do not always operate independently but may interact for greater or lesser influence. In the original model, the underlying factors are categorized as Enabling, Predisposing, and Reinforcing factors. In this adaptation, we instead use the three thematic categories of underlying factors identified in the above literature review. These underlying factors include some of the following: knowledge of caretaker about vaccination; religious or ethnic affiliation of caretakers; and willingness of health workers to provide services to a cluster of families of a certain ethnic group. Describing the potential factors and pathways leading to pockets of unvaccinated children following a mass vaccination campaign is the purpose of this framework.

Understanding such factors can help us predict and verify whether potential pockets of susceptible children actually exist in a population if we find that a factor is clustered geographically. A geographic clustering of such a factor suggests a potential pocket of unvaccinated children. Then, if such a potential pocket of unvaccinated children is identified, we can take steps before, during, and after the campaign to prevent and/or verify the existence of a true pocket. Thus, the framework can help us frame the questions and the approach used for identifying whether pockets of unvaccinated children exist following a mass campaign.

The limitations of the literature review in this paper include the possibility that available literature was missed by the search process used. Published literature might be missing. Grey literature, such as unpublished survey reports or qualitative studies, was limited to personal communications and knowledge of the authors. Much information about this topic might be available in grey literature unknown to the authors. In addition, there are potential factors that may never have been fully studied in relation to mass immunization campaigns and, therefore, do not appear in any literature (published, unpublished, grey literature). It is the case, for example, that quantitative studies of immunization may miss (within data-collection instruments) potential factors that were identified through qualitative studies (52,53). Factors that have influenced the use of other types of health services, e.g. women's autonomy, perceptions of public vs private services or the performance of health systems, e.g. decentralization, working conditions of health workers, may also be influential in mass immunization campaigns but have never been studied in this context (54-56). In addition, other conceptual models or frameworks indicate categories of factors, e.g. structural, not studied in available evaluations of mass campaigns and, therefore, not identified on the conceptual framework developed in this paper that was based on factors identified in current publications (57).

A practical, field-based methodology for identifying and preventing geographic pockets of unvaccinated persons following a mass vaccination campaign should be developed and used for supporting planning, supervision, and evaluation of such campaigns. The methodology should first identify geographic areas with the greatest potential for becoming a pocket of unvaccinated persons within a population of interest, such as a district or a province or a region. If the factors identified in this paper are clustered geographically rather than widely distributed in the population, these geographic clusters are of interest. Interviews with persons knowledgeable about the population where a campaign will be implemented, such as a district or a province, may help identify population subgroups where one or more factors identified in this paper are clustered rather than widely distributed in catchment area of the campaign.

The factors identified in this paper can provide the partial content of an interview checklist. Because of the potential limitations of this paper described above, the methodology should be supplemented with other information, such as surveillance data. Review of existing data from the past campaigns may indicate pockets in the past that are potential pockets in upcoming campaigns. Information from surveillance systems about recent outbreaks of vaccine-preventable diseases may indicate potential pockets of unvaccinated persons. Also, allowing for open-ended questions about potential pockets with local experts may indicate factors not previously studied in this context. In summary, the first step in the proposed method is to identify areas where the factors identified in this paper, along with other indications of the past or current problems, are clustered geographically versus widely distributed within a population of interest.

The proposed methodology should next include a desk review of mass campaign plans for sites identified as potential pockets in the previous step. If problems in the planning for these sites are identified, these problems can be addressed prior to the start of the campaign. The third step should be to give these sites priority for supervision during implementation of the campaign. The fourth step of the proposed method should be a post-campaign evaluation in as many sites as possible where potential pockets exist. The purpose of the post-campaign phase is to confirm (or disprove) hypotheses that one or more geographic areas contain a pocket of unvaccinated children. Information about potential pockets needing evaluation in the post-campaign phase may come from information gathered before, during, or after the campaign, e.g. by supervisors' reports. In summary, if potential pockets of unvaccinated persons are identified before, during, or after a campaign, steps can also be taken to prevent or address the problems before, during, or after the campaign. Note that the information needed to develop appropriate solutions may require further investigation before action can be taken. The method as proposed here may provide sufficient information for identifying an actual or potential pocket but may not provide all the information needed to act on the problem.
